# Radical containing combustion derived particulate matter enhance pulmonary Th17 inflammation via the aryl hydrocarbon receptor

**DOI:** 10.1186/s12989-018-0255-3

**Published:** 2018-05-03

**Authors:** Sridhar Jaligama, Vivek S. Patel, Pingli Wang, Asmaa Sallam, Jeffrey Harding, Matthew Kelley, Skylar R. Mancuso, Tammy R. Dugas, Stephania A. Cormier

**Affiliations:** 1 Department of Pediatrics, University of Tennessee Health Science Center, Memphis, TN 38103 USA; 2 Children’s Foundation Research Institute, Le Bonheur Children’s Hospital, Memphis, TN 38103 USA; 3 Department of Biological Sciences, Louisiana State University, Baton Rouge, LA 70803 USA; 4 Department of Comparative Biomedical Sciences, Louisiana State University School of Veterinary Medicine, Room 2510, 1909 Freight Dock, Skip Bertman Drive, Baton Rouge, LA 70803 USA; 5 Department of Respiratory and Critical Care Medicine, Second Affiliated Hospital, Zhejiang University School of Medicine, Hangzhou, China; 6 Department of Pharmacology, Toxicology, and Neuroscience, Louisiana State University Health Sciences Center, Shreveport, LA 71103 USA; 7 St. Joseph’s Academy, Baton Rouge, LA 70808 USA

**Keywords:** Free radicals, Particulate matter, Th17, Inflammation, Asthma, Aryl hydrocarbon receptor, Neutrophils

## Abstract

**Background:**

Pollutant particles containing environmentally persistent free radicals (EPFRs) are formed during many combustion processes (e.g. thermal remediation of hazardous wastes, diesel/gasoline combustion, wood smoke, cigarette smoke, etc.). Our previous studies demonstrated that acute exposure to EPFRs results in dendritic cell maturation and Th17-biased pulmonary immune responses. Further, in a mouse model of asthma, these responses were enhanced suggesting exposure to EPFRs as a risk factor for the development and/or exacerbation of asthma. The aryl hydrocarbon receptor (AHR) has been shown to play a role in the differentiation of Th17 cells. In the current study, we determined whether exposure to EPFRs results in Th17 polarization in an AHR dependent manner.

**Results:**

Exposure to EPFRs resulted in Th17 and IL17A dependent pulmonary immune responses including airway neutrophilia. EPFR exposure caused a significant increase in pulmonary Th17 cytokines such as IL6, IL17A, IL22, IL1β, KC, MCP-1, IL31 and IL33. To understand the role of AHR activation in EPFR-induced Th17 inflammation, A549 epithelial cells and mouse bone marrow-derived dendritic cells (BMDCs) were exposed to EPFRs and expression of *Cyp1a1* and *Cyp1b1,* markers for AHR activation, was measured. A significant increase in *Cyp1a1* and *Cyp1b1* gene expression was observed in pulmonary epithelial cells and BMDCs in an oxidative stress and AHR dependent manner. Further, in vivo exposure of mice to EPFRs resulted in oxidative stress and increased *Cyp1a1* and *Cyp1b1* pulmonary gene expression. To further confirm the role of AHR activation in pulmonary Th17 immune responses, mice were exposed to EPFRs in the presence or absence of AHR antagonist. EPFR exposure resulted in a significant increase in pulmonary Th17 cells and neutrophilic inflammation, whereas a significant decrease in the percentage of Th17 cells and neutrophilic inflammation was observed in mice treated with AHR antagonist.

**Conclusion:**

Exposure to EPFRs results in AHR activation and induction of *Cyp1a1* and in vitro this is dependent on oxidative stress. Further, our in vivo studies demonstrated a role for AHR in EPFR-induced pulmonary Th17 responses including neutrophilic inflammation.

**Electronic supplementary material:**

The online version of this article (10.1186/s12989-018-0255-3) contains supplementary material, which is available to authorized users.

## Background

Combustion of hazardous waste and biofuels produces many atmospheric pollutants including reactive trace gases, polycyclic aromatic hydrocarbons, and particulate matter (PM) [1, 2]. It is estimated that about 40–70% PM is attributed to emissions from combustion and thermal remediation sources [3]. Several epidemiological studies have provided substantial evidence that long-term exposure to PM contribute to the development of asthma and aggravate existing asthma symptoms [4–6]. Although several studies including our own studies have demonstrated an association between exposure to PM derived from combustion sources and exacerbation of asthma and respiratory tract infections [7, 8], very limited data exists about the underlying mechanisms of their toxicity and the deteriorating effects leading to the development of respiratory diseases as a result of their complexity and non-uniformity in composition. Recent studies have identified environmentally persistent free radicals (EPFRs) in PM from a variety of combustion sources including thermal remediation of hazardous wastes, diesel/gasoline combustion, wood smoke, cigarette smoke, etc. [9–14]. In the current study, we utilized an EPFR-containing ultrafine pollutant particle system that was created by exposure of PM surrogates (CuO on silica substrate) to 2-monochlorophenol vapors at 230 °C (MCP230) [15, 16]. This procedure has been shown to mimic the formation of EPFRs in the cooling zone of combustion systems. It represents the chlorinated phenol containing surface stabilized semi-quinone type free radical emitted as the effluent from a variety of combustion sources including biomass fuels, fossil fuels, and hazardous materials where chlorinated hydrocarbons and Cu(II)O are typically present [16, 17].

Our previous studies demonstrated that acute exposure of mice to EPFRs resulted in cytotoxicity, pulmonary oxidative stress, and lung dysfunction [8, 15]. Lung dysfunction correlated with increased maturation of dendritic cells and a Th17-biased immunophenotype in the lungs, however the mechanism of these responses was not completely understood. IL17A and Th17 cells have been shown to play an important role in the pathophysiology of airway diseases such as asthma and COPD [18, 19]. Cellular infiltration involving neutrophils and eosinophils is a characteristic feature of chronic inflammatory diseases [20]. Further, exaggerated neutrophilic inflammation and Th17 immune responses are hallmarks of severe asthma and are implicated in the development and promotion of steroid-resistant asthma [18, 21–23]. Th17 effector cytokines such as IL17A recruit neutrophils into the airway [24, 25]. These data warranted the investigation of underlying molecular pathways leading to EPFR-induced IL17A and Th17 responses.

Aryl hydrocarbon receptor (AHR) is a ubiquitous ligand-dependent transcription factor [26]. AHR binds to a wide array of exogenous and endogenous ligands resulting in the induction of xenobiotic metabolizing enzymes. Several researchers have demonstrated induction of cytochrome P450 metabolizing enzymes such as Cyp1A1 as a result of environmental exposure to known AHR ligands such as 2,3,7,8-tetrachlorodibenzo-p-dioxin (TCDD). The levels of AHR and its activity are modulated by exposure to its ligands [27]. Recent studies on AHR have implicated them in induction of various cytokines and chemokines and have demonstrated the role of AHR in regulating the differentiation of Th17 cells and production of Th17 cytokines such as IL17A and IL22 [28]. Depending on the type of ligand, activation of AHR can cause either Th17 or regulatory T cell differentiation leading to exacerbation of inflammation or immunosuppression, respectively [29–31]. The role of AHR in PM-induced pulmonary Th17 inflammation has not been well studied.

In the current study, we determined the mechanism underlying EPFR-induced Th17 inflammation. We demonstrated that Th17 cells are essential for EPFR-induced pulmonary neutrophilic inflammation and cytokine response. Further, we demonstrated that exposure to EPFRs results in AHR activation, as evidenced by increased expression of AHR dependent genes *Cyp1a1* and *Cyp1b1*, which is dependent on EPFR-induced oxidative stress and cellular uptake of the particles. Inhibition of AHR nuclear translocation and DNA binding abated EPFR-induced pulmonary Th17 immune responses and associated neutrophilic inflammation. Thus, EPFR-induced Th17 inflammatory responses are dependent on AHR activation.

## Methods

### Animals

Male and female C57BL/6 mice (age 8–10 weeks) were purchased from Harlan (Indianapolis, IN). Male Ahr knockout (Ahr^−/−^; B6.129-*Ahr*^*tm1Gonz*^/Nci) mice (age 16 weeks) were obtained from National Cancer Institute (Frederick, MD). The IL17Ra^−/−^ (*Il17ra*^*tm1Koll*^) and IL23p19^−/−^ (*Il23a*^*tm1Lex*^) on C57BL/6 background were obtained from Amgen Inc. and Taconic, respectively. Both male and female mice (age 8–10 weeks) were used for experiments involving IL17Ra^−/−^ and IL23p19^−/−^ mice. All mice were given free access to rodent chow and water ad libitum and were maintained under controlled conditions with 12 h light/dark cycle, temperature, humidity, and specific pathogen free conditions. All animal protocols were prepared according to the *Guide for the Care and Use of Laboratory Animals*, and were approved by the Institutional Animal Care and Use Committee at the University of Tennessee Health Science Center and Louisiana State University Health Sciences Center. Time-points were chosen to capture peak antigen presenting cell (hours) and Th cell responses (generally peak within a week after initial antigen stimulation) and reduce animal numbers needed to observe these responses.

### Exposure to PM and treatment with AHR antagonist

Radical containing ultrafine PM with mean aerodynamic diameter of approximately 0.2 μm was previously characterized by our colleague Dr. Slawo Lomnicki at the Louisiana State University as described earlier [16]. MCP230 particles were suspended in sterile saline containing 0.02% tween 80 (particle solution) at 1 mg/mL concentration. The resulting suspension was subjected to probe sonication on ice to disperse the particles and maintain a suspension free of particulate aggregates. Mice received 50 μl of the resulting suspension via oropharyngeal aspiration as described earlier [32]. Control mice were administered 50 μl of particle solution (Vehicle). Two hours prior to exposure to vehicle or MCP230, AHR activation was blocked by treating mice with 10 mg/kg dose (i.p.) of CH223191 (1-Methyl-*N*-[2-methyl-4-[2-(2-methylphenyl) diazenyl] phenyl-1*H*-pyrazole-5-carboxamide) (Cayman Chemical, Ann Arbor, MI), which was shown to be a selective antagonist of AHR [33, 34]. Here after, CH223191 will be referred as AHR antagonist.

### Luciferase assay for AHR activation

A549 cells were transfected with both a DRE-luciferase reporter for AHR activation and a Renilla luciferase reporter (Dual-Luciferase Reporter Assay System, Promega). After 24 h, the cells were exposed to 50 μg/cm^2^ particles or 50 nM TCDD with or without 100 μM Trolox, an antioxidant. Four hours after exposure to PM, firefly luciferase activity was measured as increase in luminescence in the presence of luciferin. Renilla luciferase activity, assessed as increase in luminescence in the presence of coelenterazine, was used to normalize the AHR activation data for differences in transfection efficiencies and cell number.

### *Cyp1a1* and *Cyp1b1* expression in vitro

Human lung epithelial cells (A549) were cultured in growth medium consisting of DMEM, 10% heat-inactivated fetal bovine serum (FBS) and 100 U-mg/mL penicillin-streptomycin. Cells were plated at a density of 4 × 10^4^ cells/cm^2^ in 6-well plates and incubated for 24 h to achieve ~ 80–85% confluence. Treatment groups included media-only control; vehicle control (2.7% DMSO); antioxidant 10 mM *N*-tert-Butyl-α-phenylnitrone (PBN); uptake blocker cocktail (2.5 μg/mL Filipin III; 10 μg/mL chlorpromazine; 10 μM Wortmannin); 50 μg/cm^2^ MCP50 (non-EPFR-containing particles); 50 μg/cm^2^ MCP230; MCP230+PBN; MCP50+uptake blocker cocktail; MCP230+uptake blocker cocktail. Cells receiving both particles and PBN and/or uptake blocker cocktail were pre-treated with PBN and/or uptake blocker cocktail for one hour before exposure to particles as described previously [35]. Each group was done in triplicate and cells were incubated for 4 h at 37 °C and 5% CO_2_. At the end of incubation, supernatants were collected and flash frozen in liquid nitrogen. Cells were washed twice with ice-cold PBS, scraped off the plate using a rubber policeman, pelleted and flash frozen in liquid nitrogen and stored at − 80 °C.

### Quantitative real time PCR

Total RNA was isolated using RNeasy Plus Mini Kit (Qiagen, Valencia, CA) following manufacturer’s instructions and cDNA was synthesized using Superscript III First Strand Synthesis Supermix as per manufacturer’s instructions (Life Technologies, Carlsbad, CA). 50 ng of cDNA was mixed with Power SYBR Green PCR Master Mix and qPCR was performed using Roche LightCycler 480, (Applied Biosystems, Foster City, CA). Primers sequences for *Cyp1a1* (Amplicon Length: 148; Forward: GAGGAGCTAGACACAGTGATTG; Reverse: TGTCTCTTGTTGTGCTGTGG); *Cyp1b1* (Amplicon Length: 151; Forward: CACCAGGTATCCTGATGTGC; Reverse: AGGCACAAAGCTGGAGAAG); *Hprt1* (Amplicon Length: 181; Forward: TGGCGTCGTGATTAGTGATG; Reverse: ACAGAGGGCTACAATGTGATG). *Il17a* expression was determined using TaqMan gene expression assay (Applied Biosystems, Waltham, MA). Data are normalized for *Hprt1 or Gapdh* and plotted as relative gene expression using ΔΔCt analysis.

### Bronchoalveolar lavage fluid (BALF) cellularity

Mice were humanely euthanized, and a small incision was made in the upper region of the trachea and an 18-gauge cannula was inserted into the incision. 1 mL of BALF isolation buffer (PBS containing 0.5% BSA) was slowly instilled and removed from the lungs. Cells were counted and 20,000 cells were spun on to a glass slide using a cytospin. The slides were air dried and subsequently stained with Hema-3 staining kit (Fisher Scientific, Pittsburgh, PA) following supplier’s instructions. Differential cell counts were determined based on the morphology and staining of the cells by counting at least 300 cells per slide.

### Establishment of mouse model of asthma

A mouse model of asthma using chicken egg white ovalbumin (OVA) (Sigma-Aldrich, St. Louis, MO) was generated. Briefly, a mixture of 20 μg of OVA emulsified in Imject Alum (Pierce, Rockford, IL) was prepared and mice were sensitized by injecting with the mixture i.p. on protocol days 0, and 14. MCP230 (50 μg) was administered to wild type and IL17Ra^−/−^ OVA+MCP230 group mice on protocol day 23. Subsequently, mice were challenged with 1% OVA solution made in saline by inhalation exposure for 20 min on protocol days 24, 25, and 26. Mice were euthanized on protocol day 28; BALF was collected for differential cell count analysis and lungs were collected for performing histopathological assessment.

### Lung histopathology and in situ hybridization

Mice were euthanized, lungs were isolated and inflated with zinc-formalin fixative at 25 cm constant water pressure and fixed for 24 h before they were transferred to 70% ethanol. The lungs were then dehydrated, embedded in paraffin and sectioned to 4 μm thick sections and stained with hematoxylin and eosin to visualize the inflammation. In situ hybridization for *Cyp1a1* RNA was performed as described earlier [36] using RNAscope 2.5 HD assay kit (Advanced Cell Diagnostics, Newark, CA) on lung sections of mice exposed to vehicle or MCP230. Representative images of lung sections were acquired using EVOS FL Auto cell imaging system (Life Science Technologies, Grand Island, NY). To quantify inflammation, ten random airway microscopy fields were captured at 10× magnification on the Nikon Eclipse Ci-L (Nikon Corporation, Tokyo, Japan). Area of inflammation was quantified using ImageJ software (National Institutes of Health, Bethesda, MD). Data are represented as the area of inflammation surrounding airway and normalized to the size of the respective airway.

### Flow cytometry

Single cell suspension of the lung cells and flow cytometric staining of cells was performed as described earlier [7, 32]. Mice were euthanized; lungs were subjected to retrograde vascular perfusion with Hank’s Balanced Salt solution (HBSS) to remove excess red blood cells. The isolated lungs were coarsely dissociated using an Octodissociator (Miltenyi, Germany). The dissociated lungs were incubated with collagenase I (Invitrogen, NY), and 150 ng/mL DNase I (Sigma Aldrich, MO) for 30 min in HBSS. Following incubation, the lungs were further subjected to dissociation with Octodissociator to reduce the remaining cell clumps to single cell suspension. Cells were strained through a 40 μm cell strainer (BD Biosciences, CA). The resulting cell suspension was treated with RBC lysis buffer to remove any residual blood cells. Cells were incubated for five hours at 37 °C with a stimulatory cocktail containing 5% heat-inactivated fetal bovine serum, 500 ng/mL ionomycin, 5 ng/mL phorbol 12-myristate 13-acetate (PMA) (Sigma-Aldrich), and a protein transport inhibitor (GolgiPlug, BD Biosciences) made in RPMI 1640 media. Cells were stained with a Fixable live/dead dye eFluor 780 (eBiosciences, San Diego, CA) and were subsequently fixed, permeabilized and stained for T cell and intracellular cytokine markers using antibodies eFluor450-CD3 (eBiosciences, CA), PerCP-CD4 (BioLegend, CA), FITC-CD8 (eBiosciences, CA), and PE-IFNγ (eBiosciences, CA), PE-Cy7-IL4 (eBiosciences, CA), APC-IL17A (eBiosciences, CA). A total of 1.2 million cells/sample were analyzed by FACS Canto II (BD Biosciences) and dead cells were excluded from analysis. Flow cytometry data was analyzed using FlowJo Software v.10 (FLOWJO LLC, OR).

### Cytokine analysis

Cytokine levels in homogenized lung supernatants were determined using MILLIPLEX MAP Mouse Th17 cytokine magnetic bead panel (Millipore, Billerica, MA) using Luminex 200 system (Luminex Corporation). The following cytokines were assayed: IL1β, IL6, IL17A, IL17E, IL21, IL22, IL31, IL33, keratinocyte-derived chemokine (KC) and monocyte chemotactic protein (MCP-1). Raw data were plotted against a standard curve using a five-parameter logistic regression to derive the concentrations for unknown samples. Data presented exclude numbers beyond the sensitivity of the assay.

### Statistics

Data are presented as means ± SEM. Data are analyzed by GraphPad Prism software (Version 6). ANOVA with post hoc analysis was performed to determine the level of difference between experimental groups. *p* < 0.05 was considered as statistically different.

## Results

### EPFRs induce pulmonary Th17 immune responses

**Fig. 1:**
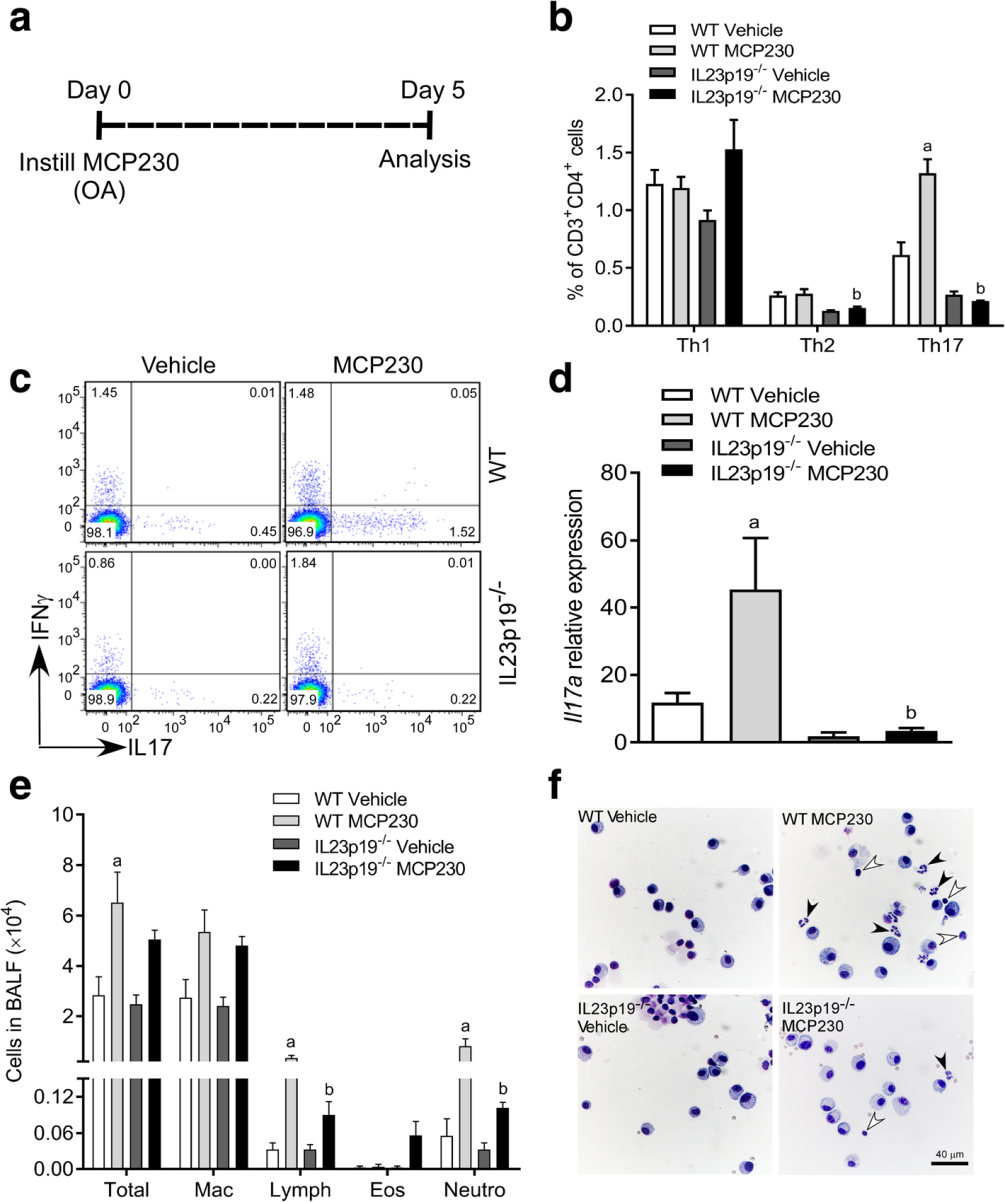
Exposure to MCP230 induces immune responses in the lung in Th17 dependent manner. (**a**) Schematic of MCP230 exposure protocol. Mice were exposed to MCP230 (50 μg) by oropharyngeal aspiration on day 0 and BAL fluid (BALF) or whole-lungs were collected on day 5 post-exposure. (**b**) T cell subsets were quantified using flow cytometry. Pulmonary T helper cell responses were measured after vehicle or MCP230 exposure in WT and IL23p19^−/−^ mice. PMA/ionomycin-stimulated lung cells were stained with surface (CD3, CD4, and CD8) and intracellular (IFNγ, IL4, and IL17A) antibodies for Th1, Th2, and Th17 cells. (**c**) Representative pseudo color dot plots of CD4^+^ T cells gated for IFNγ, and IL17A in WT and IL23p19^−/−^ mice. (**d**) The expression of *Il17a* relative to *Gapdh* in whole-lung homogenates of WT and IL23p19^−/−^ mice exposed to vehicle or MCP230 was determined using RT-qPCR. **e** Differential cell count of BALF from vehicle or MCP230-exposed WT or IL23p19^−/−^ mice. **f** Representative photomicrographs of BALF cells collected from WT and IL23p19^−/−^ mice exposed to vehicle or MCP230. Arrowheads represent the neutrophils (black) and lymphocytes (white). Data represent mean ± SEM from 3 to 5 mice. ^a^*p* < 0.05, compared to WT Vehicle group. ^b^*p* < 0.05, compared to WT MCP230 group, one-way ANOVA with Tukey’s multiple comparisons test

Our previous study demonstrated that acute exposure to EPFRs results in Th17-biased pulmonary inflammation with increased neutrophils [35]. IL23 serves as a survival and maintenance factor for Th17 cells [37, 38] and promotes Th17 differentiation [39, 40]. IL23p19^−/−^ mice are deficient in Th17 cells and, therefore, this mouse model was used to confirm that Th17 cells mediate the increase in lung IL17A and neutrophil recruitment upon MCP230 exposure. We exposed wild type (WT) and IL23p19^−/−^ mice to either vehicle or MCP230 (EPFR). Percent Th17 cells and *Il17a* expression were measured. A schematic for the MCP230 exposure and analysis of Th17 cells, *Il17a* expression and cell differential in BALF at 5 days post-exposure (dpe) is presented in Fig. 1a. In congruence with our previously published results, a significant increase in the percent of pulmonary Th17 cells was observed in WT mice exposed to MCP230 compared to vehicle. On the contrary, MCP230-exposed IL23p19^−/−^ mice failed to show an increase in the percent of pulmonary Th17 cells (Fig. 1b and c; CD3^+^CD4^+^ cells double positive for IFNγ and IL4 or IL4 and IL17A were excluded while gating). A significant increase in the expression of *Il17a* was observed in MCP230-exposed WT mice compared to vehicle, but no difference was seen in IL23p19^−/−^ mice (Fig. 1d), suggesting that increased *Il17a* expression in MCP230-exposed WT mice is Th17 dependent. Expression of *Il17f* was less than that of *Il17a* and there was no difference between MCP230-exposed WT mice compared to vehicle or to MCP230-exposed IL23p19^−/−^ mice (Additional file 1: Figure S1).

To determine the role of Th17 cells in EPFR-induced pulmonary neutrophilic inflammation, we performed differential cell counts in WT and IL23p19^−/−^ mice exposed to either vehicle or MCP230. In WT mice, exposure to MCP230 significantly increased the numbers of lymphocytes and neutrophils compared to that in vehicle control, whereas these numbers were significantly less in MCP230-exposed IL23p19^−/−^ mice compared to that in MCP230-exposed WT mice (Fig. 1e). Exposure to MCP230 in both WT and IL23p19^−/−^ mice resulted in increases in the total number of leukocytes and macrophages compared to respective vehicle exposed mice. The numbers of total leukocytes and macrophages were comparable between WT and IL23p19^−/−^ mice following exposure to MCP230 (Fig. 1e). Representative images of higher numbers of neutrophils and lymphocytes (black and white arrowheads, respectively) in MCP230-exposed WT mice compared to MCP230-exposed IL23p19^−/−^ mice are shown in Fig. 1f.

### EPFRs induce pulmonary neutrophilic inflammation in an IL17A-dependent manner

**Fig. 2:**
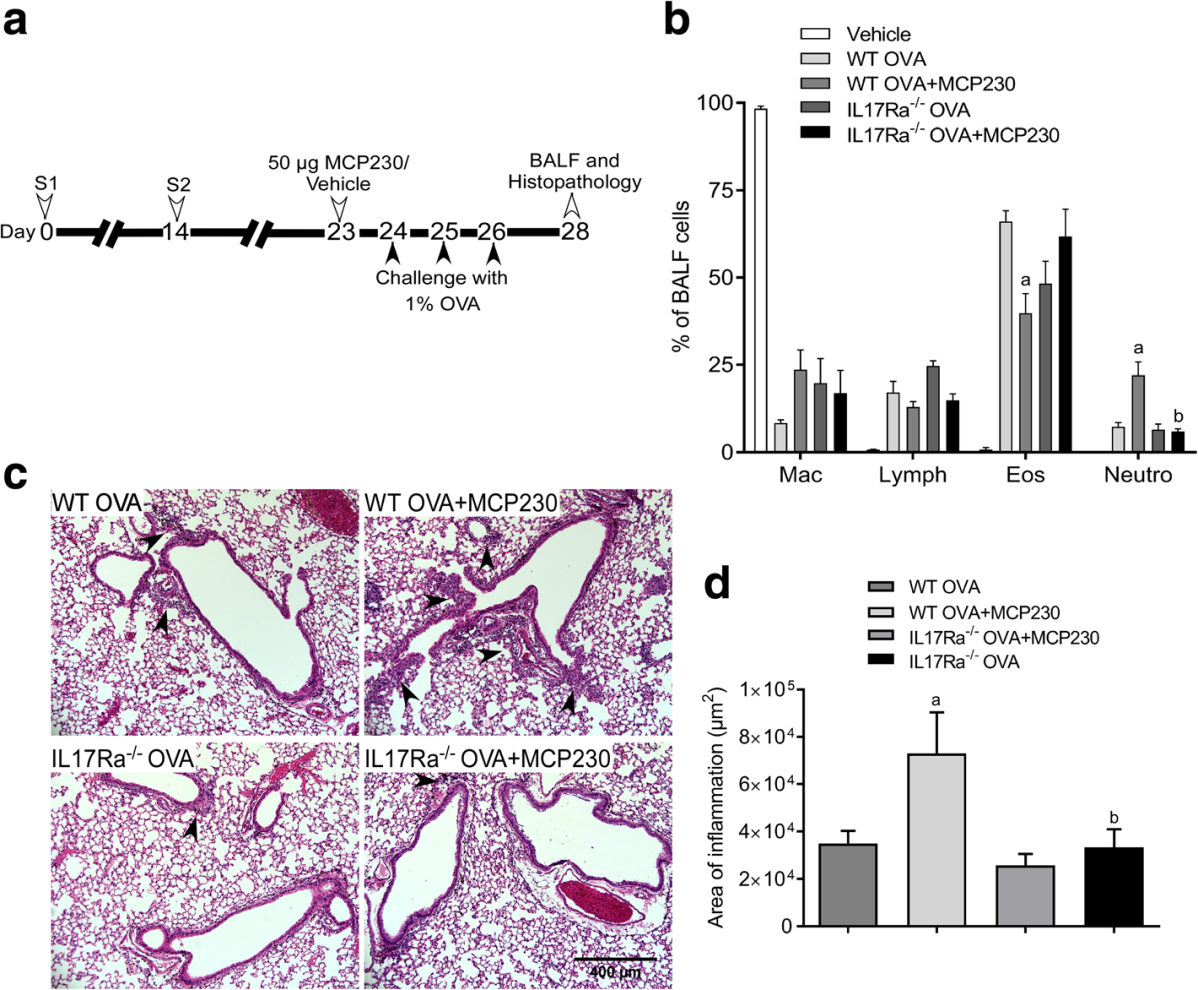
IL17A mediates MCP230-exacerbated pulmonary neutrophilic inflammation in asthma. **a** Schematic representing the protocol followed to induce inflammation in a mouse model of asthma. WT and IL17Ra^−/−^ mice were sensitized with OVA (Ovalbumin complexed to Imject Alum) on days 0 and 14. Mice were exposed to either vehicle or MCP230 (50 μg) on protocol day 23 and challenged with OVA on days 24, 25, and 26. BALF or lungs were collected on day 28. **b** Differential cell counts of BALF cells at 5 days post-exposure (i.e. day 28) from mice challenged with OVA and exposed to vehicle or MCP230. Data are represented as percentage of total BALF cells. Data represent mean ± SEM from 4 to 5 mice. ^a^p < 0.05, compared to WT OVA group. ^b^*p* < 0.05, compared to WT OVA+MCP230 group, one-way ANOVA with Holm-Sidak’s multiple comparisons test. **c** Representative photomicrographs of the lungs of WT and IL17Ra^−/−^ mice challenged with OVA and exposed to vehicle or MCP230. Arrowheads point to areas of peribronchiolar and perivascular inflammation. **d** Quantification of area of inflammation (μm^2^) surrounding the airways. Data represent mean ± SEM from 4 to 5 mice. ^a^*p* < 0.05, compared to WT OVA group. ^b^*p* < 0.05, compared to WT OVA+MCP230, one-way ANOVA with Tukey's multiple comparisons test

To demonstrate that MCP230 exacerbates asthma and determine if MCP230-induced pulmonary neutrophilic inflammation is dependent on IL17A, an OVA-induced mouse model of asthma was developed in WT and IL17 receptor alpha knockout (IL17Ra^−/−^) mice. A schematic for the development of the mouse model of asthma, exposure to MCP230, and BALF and histopathology assessment is presented in Fig. 2a. BALF was collected for differential cell counts and pulmonary inflammation was determined by hematoxylin and eosin staining of lung sections obtained at 5 dpe. A significant increase in the percentage of neutrophils was observed in WT mice exposed to OVA and MCP230 (WT OVA+MCP230) compared to WT OVA mice; however, the percentage of neutrophils was significantly less in IL17Ra^−/−^ OVA+MCP230 mice compared to MCP230-exposed WT mice (Fig. 2b). No difference in the percentage of neutrophils was observed in IL17Ra^−/−^ OVA+MCP230 mice compared to IL17Ra^−/−^ OVA mice. The percentage of eosinophils was significantly decreased in WT OVA+MCP230 mice compared to WT OVA mice (Fig. 2b); and there were no significant changes in the numbers of macrophages and lymphocytes. Total numbers of cells are shown in Additional file 2: Figure S2. Histopathological analysis revealed inflammation in the peribronchiolar/perivascular areas in lungs of WT OVA+MCP230 mice, while such inflammation was absent or mild in IL17Ra^−/−^ OVA+MCP230 mice (Fig. 2c and d). Together, these results suggest that MCP230-induced pulmonary neutrophilic inflammation in WT OVA+MCP230 mice is dependent on IL17A.

### Exposure to EPFRs induces activation of AHR

**Fig. 3:**
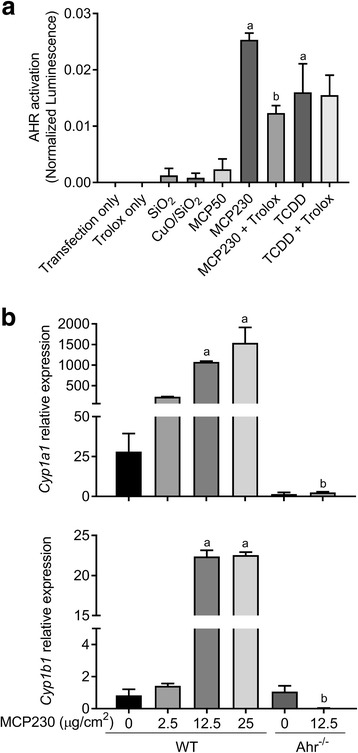
MCP230 exposure activates aryl hydrocarbon receptor (AHR) and increases the expression of *Cyp1a1.* (**a**) Activation of AHR as measured using a dual AHR luciferase reporter assay in A549 cells. A549 cells were exposed to MCP230 (50 μg/cm^2^) or TCDD (50 nM) and simultaneously treated with or without trolox (antioxidant; 100 μM) for 4 h. In addition, AHR activation was assessed in cells exposed to non-EPFR-containing particle controls such as SiO_2_, CuO/SiO_2_, and MCP50 (50 μg/cm^2^). AHR promoter activity is expressed as normalized luminescence using a Renilla reporter for internal normalization. Data represent mean ± SEM from one of two independent experiments, performed in triplicate. ^a^*p* < 0.05, compared to MCP50 group. ^b^*p* < 0.05, compared to MCP230 group, one-way ANOVA with Dunnett’s multiple comparisons test. (**b**) Activation of AHR as measured by *Cyp1a1* and *Cyp1b1* expression relative to *Gapdh* using RT-qPCR analysis in the bone marrow-derived dendritic cells (BMDCs). BMDCs from WT and Ahr^−/−^ mice were exposed to various concentrations of MCP230 for 4 h. Data represent mean ± SEM from one of two independent experiments, performed in duplicate. ^a^*p* < 0.05, compared to WT (0 μg/cm^2^) group. ^b^*p* < 0.05, compared to WT (12.5 μg/cm^2^) group, one-way ANOVA with Tukey’s multiple comparisons test

AHR binds to a wide array of exogenous ligands (e.g. air pollutants) resulting in the induction of xenobiotic metabolizing enzymes and cytokines. Depending on the type of ligand, AHR regulates differentiation of Th17 cells and production of Th17 cytokines such as IL17A and IL22 [28]. To understand the role of AHR in MCP230-induced Th17 inflammation, we investigated whether acute exposure to MCP230 activates the AHR pathway. AHR activation was analyzed in vitro using a dual luciferase reporter assay in MCP230-exposed A549 cells. MCP230 exposure caused a significant increase in luciferase activity as measured by luminescence, indicating AHR activation in MCP230-treated cells compared to that of MCP50 (PM containing the organic and particle but lacking the EPFR [16]) treated cells (Fig. 3a). The increase in the luminescence was comparable to the luminescence in TCDD-exposed cells, a known AHR agonist. A significant reduction in AHR activation was observed when MCP230-exposed cells were co-treated with trolox, an antioxidant. However, such reduction was not observed in TCDD exposed cells co-treated with trolox. No significant increase in AHR activation was observed in cells treated with non-EPFR-containing PM (SiO_2_, CuO/SiO_2_, or MCP50) compared to transfection only group. These observations demonstrate that EPFRs are important for the activation of AHR.

Using in vitro and in vivo studies, we previously demonstrated that MCP230 induces the maturation of DCs and enhances the capacity of DCs to stimulate the proliferation and activation of T cells [35]. Recently, it was reported that AHR activation also induces maturation of DCs [41]. To assess the role of MCP230 to activate AHR in DCs cuing Th17 responses and subsequent neutrophilia, we determined the expression of AHR regulated genes *Cyp1a1* and *Cyp1b1* in bone marrow-derived dendritic cells (BMDCs) isolated from WT and Ahr^−/−^ mice. Treatment of BMDCs derived from WT mice with MCP230 caused a dose-dependent increase in the expression of *Cyp1a1* with significant difference at concentrations 12.5 and 25 μg/cm^2^ compared to vehicle treated control cells (Fig. 3b). Also, a significant increase in the expression of *Cyp1b1* was observed with exposure to MCP230 at same concentrations. In contrast, and as expected, treatment of BMDCs isolated from Ahr^−/−^ mice with MCP230 at 12.5 μg/cm^2^ did not result in any change in *Cyp1a1* and *Cyp1b1* expression compared to respective vehicle treated control cells (Fig. 3b). Together, these data indicate that exposure to MCP230 induces AHR activation.

### EPFRs induce expression of *Cyp1a1* and *Cyp1b1* in vitro in an uptake-dependent and oxidative stress-dependent manner

**Fig. 4:**
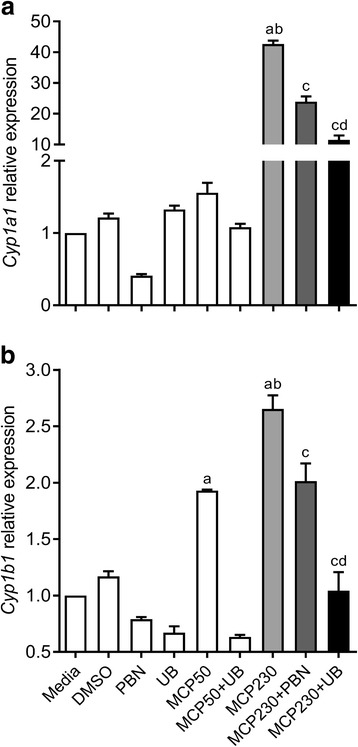
Exposure to MCP230 increases the expression of *Cyp1a1* and *Cyp1b1* in an uptake- and oxidative stress-dependent manner. A549 cells were exposed to MCP230 (50 μg/cm^2^) and simultaneously treated with an antioxidant (PBN; 10 mM) or an uptake blocker cocktail (UB) for 4 h. *Cyp1a1* (**a**) and *Cyp1b1* (**b**) expression relative to *Hprt* was determined by RT-qPCR. Data represent mean ± SEM from one of two independent experiments, performed in triplicate. ^a^*p* < 0.05, compared to Media group. ^b^*p* < 0.05, compared to MCP50 group. ^c^*p* < 0.05, compared to MCP230 group. ^d^*p* < 0.05, compared to MCP230+PBN group, one-way ANOVA with Tukey’s multiple comparisons test

Previously, we reported that blocking cellular uptake of MCP230 or oxidative stress inhibits the MCP230-induced T cell activation and proliferation [35]. Thus, uptake of MCP230 particles or oxidative stress induced by MCP230 particles is important for its immune-modulating effects. To determine if uptake of MCP230 was necessary for the activation of AHR, we pretreated A549 cells with an uptake blocker cocktail (UB) or antioxidant PBN followed by exposure to MCP230 and measured the expression of *Cyp1a1* and *Cyp1b1*. Exposure to MCP230 significantly increased the expression of *Cyp1a1* (> 40-fold) and pretreatment with UB or PBN partially inhibited the effects of MCP230 on *Cyp1a1* expression (Fig. 4a). Expression of *Cyp1b1* was also reduced in cells treated with UB or PBN and exposed to MCP230. Although, exposure to MCP50 caused a significant increase in *Cyp1b1* expression, treatment of cells with UB significantly inhibited the expression of *Cyp1b1* (Fig. 4b). These results indicate that MCP230-induced AHR activation is dependent on both particle uptake and the ability to induce generation of reactive oxygen species (ROS).

### Exposure to EPFRs induces *Cyp1a1* and *Cyp1b1* expression in an AHR-dependent manner

**Fig. 5:**
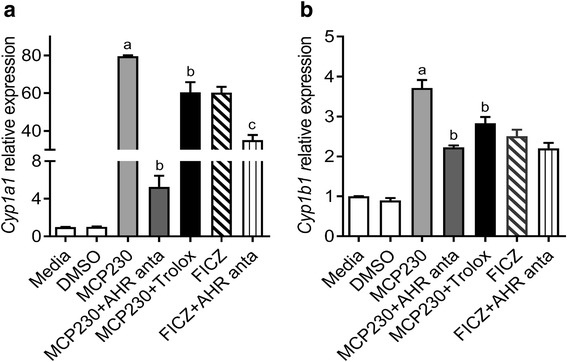
MCP230-induced expression of *Cyp1a1* and *Cyp1b1* is dependent on AHR activation. A549 cells were treated with MCP230 (50 μg/cm^2^) or AHR agonist (FICZ; 200 nM) in the presence or absence of AHR antagonist (CH223191; 10 μM) for 4 h. Cell pellets were collected and expression of *Cyp1a1* (**a**) and *Cyp1b1* (**b**) relative to *Hprt* was determined using RT-qPCR analysis. Data represent mean ± SEM from one of two independent experiments, performed in triplicate. ^a^*p* < 0.05, compared to Media group. ^b^*p* < 0.05, compared to MCP230 group. ^c^*p* < 0.05, compared to FICZ group, one-way ANOVA with Tukey’s multiple comparisons test

To determine if MCP230-induced expression of *Cyp1a1* and *Cyp1b1* was dependent on activation of AHR, we exposed A549 cells to MCP230 in the presence or absence of CH223191, a selective AHR antagonist (AHR anta) that inhibits AHR-dependent transcription even at 10 μmol concentration [34]. FICZ is a high affinity ligand of AHR and it has been shown to enhance Th17 responses and exacerbate immune-mediated diseases in several mouse models [28, 31, 42]. Since the effects of MCP230 are similar to that of FICZ, we switched to using FICZ as a positive control for AHR activation. Exposure to MCP230 and FICZ caused a significant increase in the expression of *Cyp1a1* (Fig. 5a) and *Cyp1b1* (Fig. 5b) compared to media or vehicle (DMSO) controls. Pretreatment of MCP230-exposed cells with AHR antagonist significantly reduced the expression of both *Cyp1a1* and *Cyp1b1* compared to that in cells exposed to MCP230 alone. Similarly, FICZ-induced *Cyp1a1* and *Cyp1b1* expression was also inhibited by AHR antagonist, indicating an AHR dependent expression of these proteins. These results further corroborate the data presented in Figs. 3 and 4 that demonstrate MCP230-induced activation of AHR.

### Acute exposure to EPFRs results in transient activation of AHR in vivo

**Fig. 6:**
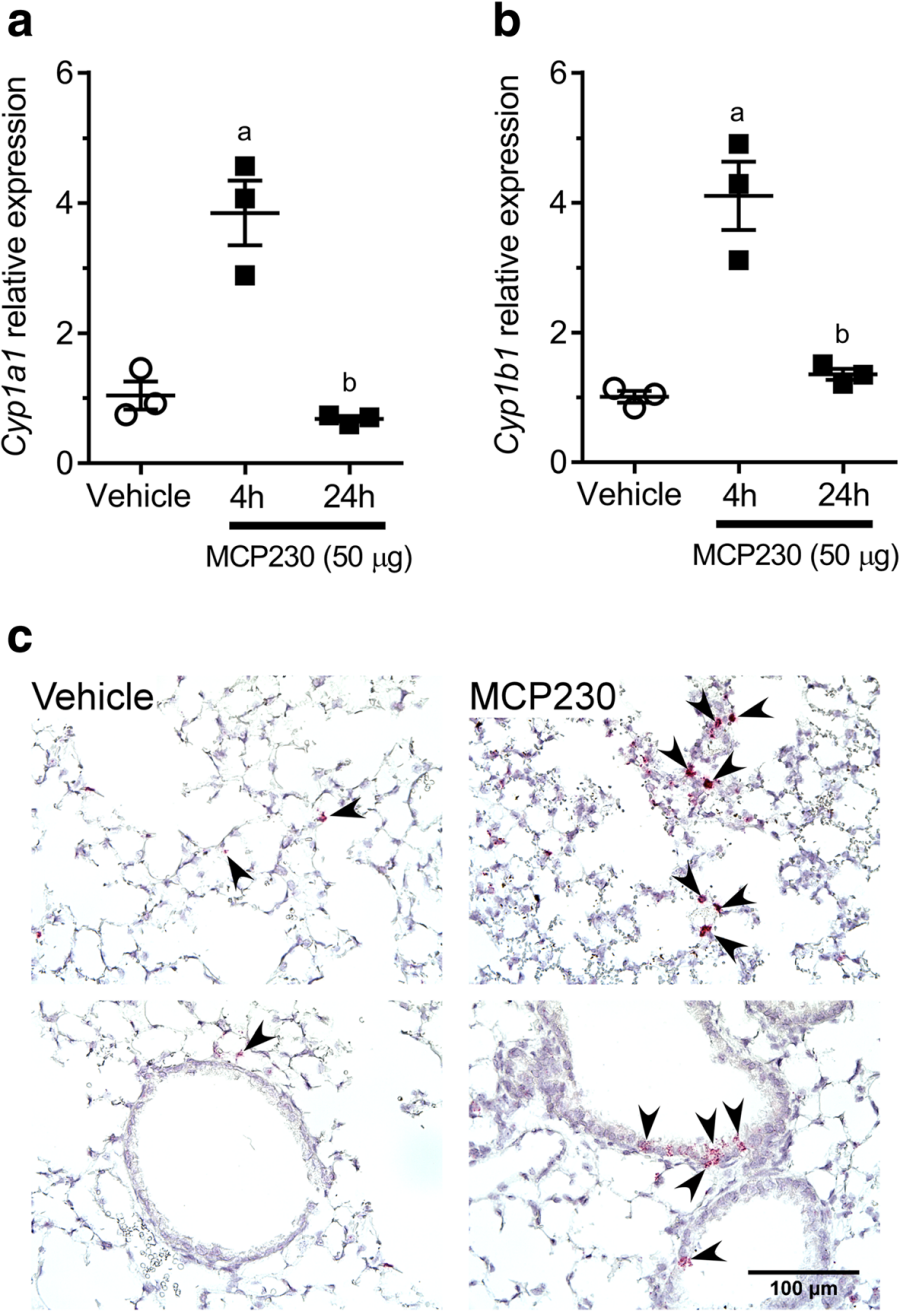
Acute exposure to MCP230 results in transient activation of AHR and increased expression of *Cyp1a1* and *Cyp1b1* in vivo. (**a**, **b**) WT mice were exposed to vehicle or MCP230 (50 μg) and lungs were isolated at 4 and 24 h post-exposure. Expression of *Cyp1a1* and *Cyp1b1* relative to *Hprt* was measured using RT-qPCR analysis. Data represent mean ± SEM from one of two independent experiments. ^a^p < 0.05, compared to Vehicle group. ^b^*p* < 0.05, compared to 4 h group, one-way ANOVA with Tukey’s multiple comparisons test. (**c**) Representative photomicrographs of lungs from WT mice exposed to vehicle or 50 μg MCP230. Lungs were isolated from mice at 4 h post-exposure and stained for *Cyp1a1* RNA using in situ hybridization. Top panel represents expression of *Cyp1a1* in lung parenchyma and bottom panel represents expression of *Cyp1a1* in the airway epithelium. Arrowheads point at cells expressing *Cyp1a1*

We investigated whether exposure to EPFRs result in transient or persistent activation of AHR in vivo*.* WT mice were exposed to vehicle or MCP230 (50 μg) via oropharyngeal aspiration, lungs were isolated at 4 and 24 h post-exposure, and expression of *Cyp1a1* and *Cyp1b1* genes was determined. Exposure to MCP230 resulted in a significant increase in the expression of *Cyp1a1* and *Cyp1b1* at 4 h post-exposure compared to vehicle treated controls (Fig. 6a and b). At 24 h post-exposure, the expression of both *Cyp1a1* and *Cyp1b1* were reduced to that of the vehicle treated control (Fig. 6a and b). These data indicate that acute exposure to MCP230 results in transient, but significant, increases in AHR activity in the lungs. To determine if AHR was activated in airway or immune cells, in situ hybridization for *Cyp1a1* mRNA was performed in the lungs isolated at 4 h post-exposure from mice exposed to vehicle or MCP230. A significant increase in *Cyp1a1* was observed in the lungs of the mice exposed to MCP230 compared to vehicle control. An increase in *Cyp1a1* positive cells was observed in the airway epithelium and in the parenchymal regions in the lungs of MCP230-treated mice (Fig. 6c). The specific cell types in which MCP230 induced *Cyp1a1* were not identified.

### Exposure to EPFRs induces Th17 cytokine response in the lung

In a different study in our lab, we observed that AHR activation is increased through to 12 h and returns to baseline by 24 h (data not shown). To determine the effects of MCP230 exposure on immediate immune responses, we measured Th17 associated cytokines in the lungs of MCP230-exposed mice at 12 h post-exposure and 1 dpe. A total of 10 cytokines (IL1β, IL6, IL21, IL17A, IL22, IL17E, KC, MCP-1, IL33 and IL31) were assessed. Acute exposure to MCP230 significantly increased the levels of Th17 polarizing cytokines IL1β and IL6 at 1 dpe, with significantly higher levels of IL6 (approximately 45 fold increase) as early as 12 h post-exposure (Table 1). Th17 effector cytokines IL17A and IL22 significantly increased at 1 dpe in the lungs of MCP230-exposed mice, but were not different at 12 h. These results indicate that exposure to MCP230 induces a Th17 cell-biased environment in the lungs. We also analyzed the chemokines KC and MCP-1 in the lungs of MCP230-exposed mice. The levels of both KC and MCP-1 were significantly elevated at 1 dpe, with significantly higher levels of MCP-1 as early as 12 h. In addition, we observed an increase in the levels of IL33 at 12 h and 1 dpe. In contrast to the IL33 levels, a reduction in the levels of IL31 was observed, which was significant at 12 h. No significant changes in the levels of IL17E and IL21 were observed in mice exposed to MCP230 compared to vehicle control (Table 1).

**Table 1:** MCP230 exposure results in pulmonary Th17 associated cytokine induction

	MCP230	
Cytokines	12 h	1 d
IL1β	1.446 ± 0.1729	1.691 ± 0.2082^a^
IL6	45.71 ± 8.457^a^	10.61 ± 3.177^a^
IL21	0.7346 ± 0.0256	1.034 ± 0.1367
IL17A	0.6526 ± 0.1302	6.096 ± 1.666^a^
IL22	0.9377 ± 0.0325	1.504 ± 0.158^a^
IL17E	0.7034 ± 0.0941	0.9023 ± 0.0322
KC	5.629 ± 1.54 ^*p = 0.053*^	2.591 ± 0.4729^a^
MCP-1	6.337 ± 1.325^a^	6.825 ± 0.2238^a^
IL33	2.226 ± 0.1384^a^	2.716 ± 0.1907^a^
IL31	0.3472 ± 0.07782^a^	0.4988 ± 0.0489

Levels of Th17 cytokines were determined in the lung homogenates of WT mice exposed to MCP230 at 12 h and 1 day post-exposure. Concentrations (pg/mg of lung protein) of all cytokines were measured using multiplex assay. Data are expressed as means ± SEM of fold change over Vehicle treated controls from 4 to 5 mice. ^a^*p* < 0.05 compared to vehicle control, unpaired t test

### EPFR-induced Th17 pulmonary immune response and pulmonary neutrophilic inflammation are dependent on AHR activation

**Fig. 7:**
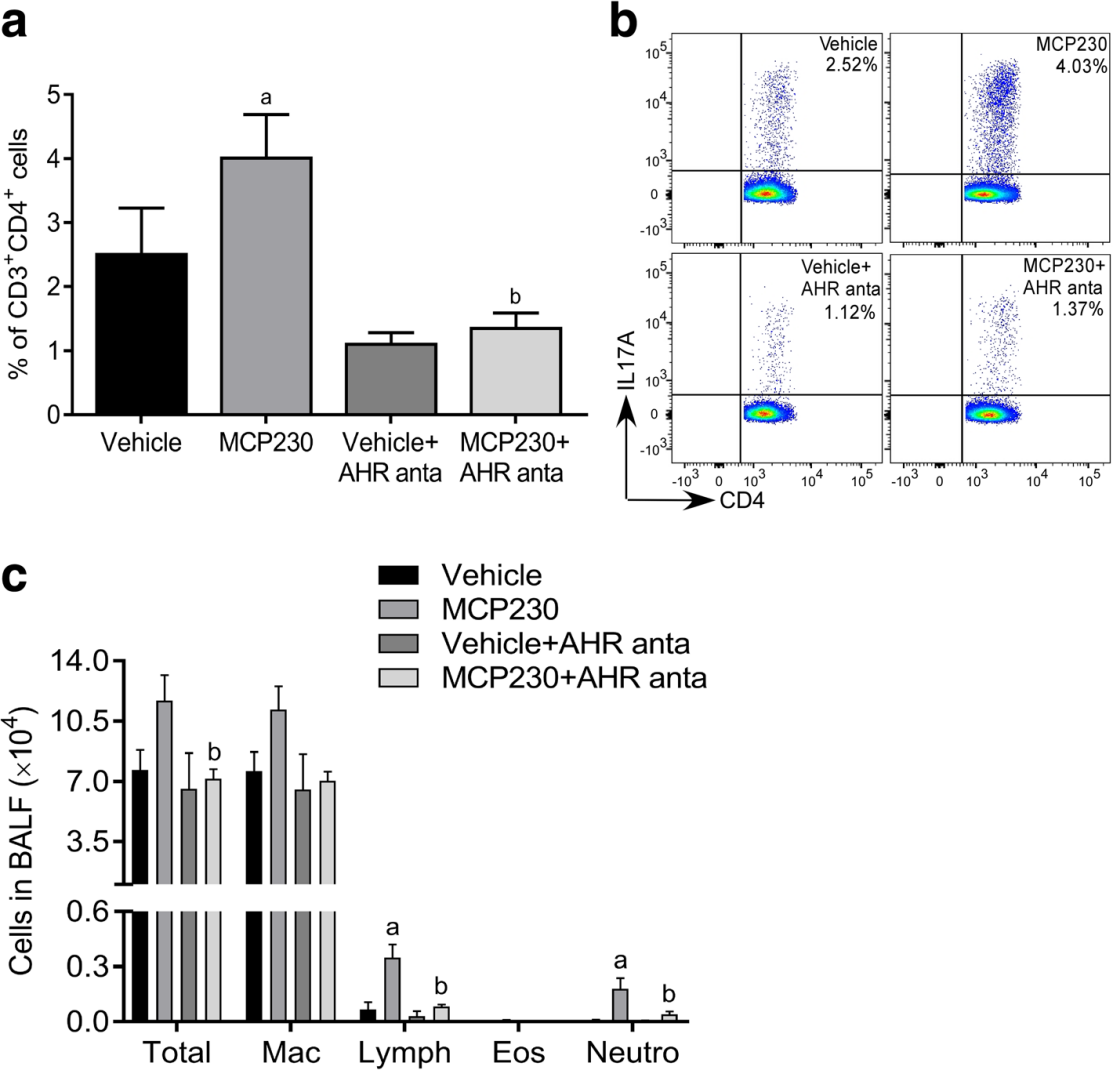
MCP230-induced pulmonary Th17 immune response is dependent on AHR activation. WT mice were exposed to vehicle or MCP230 (50 μg) that were treated with or without AHR antagonist (CH223191; 10 mg/kg) 2 h prior to the exposure. Lungs or BALF were collected at 5 dpe. (**a**, **b**) Lungs were used to determine adaptive T cell responses. PMA/ionomycin-stimulated lung cells were stained with surface (CD3 and CD4) and intracellular (IL17A) antibodies. T cell subsets were quantified using flow cytometry. Data are presented as percentage of IL17A^+^ cells among CD3^+^CD4^+^ cells (**a**) and representative flow cytometry pseudo color dot plots of IL17A^+^CD4^+^ cells gated on CD3^+^ T cells (**b**). Data represent mean ± SEM from 4 to 5 mice. ^a^*p* < 0.05, compared to Vehicle group. ^b^*p* < 0.05, compared to MCP230 group, one-way ANOVA with Holm-Sidak’s multiple comparisons test. (**c**) BALF were used to assess differential cell counts by counting at least 300 cells/sample. Data represent mean ± SEM from one of two independent experiments, 4 to 5 mice. ^a^*p* < 0.05, compared to Vehicle group. ^b^*p* < 0.05, compared to MCP230 group, one-way ANOVA with Dunnett’s multiple comparisons test

Exposure to MCP230 resulted in a significant increase in the percent of Th17 cells in the lungs of mice as shown in Fig. 1b and previously [35]. Recent reports indicate that AHR activation leads to Th17 cell differentiation thus leading to exacerbation of the inflammatory responses [29–31]. To investigate the role of AHR in the MCP230-induced Th17 response, we inhibited AHR activation in MCP230-exposed mice by treating them with AHR antagonist (MCP230+AHR anta) and measured pulmonary T cell sub-populations using intracellular cytokine staining. Exposure to MCP230 resulted in a significant increase in Th17 cells (IL17A producing CD3^+^CD4^+^ cells) in the lungs of mice exposed to MCP230 compared to vehicle exposed control mice. Treatment with AHR antagonist significantly reduced the percent of Th17 cells in the lungs of MCP230-exposed mice compared to that in MCP230-exposed mice not treated with AHR antagonist (Fig. 7a and b). There was no significant difference in Th17 numbers between vehicle exposed control group and AHR antagonist treated groups [i.e. Vehicle vs Vehicle+AHR anta (*p* = 0.17) or MCP230+AHR anta (*p* = 0.31)].

To determine the role of AHR in MCP230-induced neutrophil recruitment, we analyzed the inflammatory cells in airways (BALF) of mice pre-treated with AHR antagonist and exposed to MCP230. The mice were treated with AHR antagonist 2 h prior to MCP230 exposure to inhibit AHR activation (i.e. at 4 h post MCP230 exposure). In accordance with the data presented in Fig. 1, exposure to MCP230 increased the total number of leukocytes in the lungs, with significant increase in the number of neutrophils and lymphocytes compared to that in vehicle control. Pretreatment with AHR antagonist significantly attenuated these high numbers of total leukocytes, including neutrophils and lymphocytes, in MCP230-exposed mice (Fig. 7c). Together, these results suggest that AHR activation is required for MCP230-induced pulmonary Th17 response and recruitment of inflammatory neutrophils in lungs.

## Discussion

Several epidemiological studies have demonstrated association between exposure to airborne PM and increased risk to develop asthma and/or acute asthma exacerbations [43–45]. Although an association between elevated levels of PM and respiratory diseases exist, the underlying mechanisms of PM-induced asthma exacerbations are not completely understood. In this study, we utilized an EPFR-containing combustion-derived PM, a common form of PM found in the atmosphere, and present a unique mechanism that mediates the pulmonary immune responses as a result of exposure. The data presented here demonstrate that MCP230, an EPFR-containing PM, results in pulmonary neutrophilia that is dependent on Th17 cells and IL17A. Further, we demonstrate that AHR signaling is important for EPFR-induced Th17 adaptive immune response and pulmonary neutrophilia.

Pulmonary neutrophilic inflammation is a major characteristic of patients with severe asthma. Studies in humans with asthma and animal models of asthma demonstrate that neutrophils play an important role in the pathogenesis of severe asthma [21, 46]. Further, IL17A has been shown to play an important role in the severity of human airway diseases, such as asthma and COPD [47]. Airway inflammation in severe and persistent asthma is associated with IL17A; and Th17 cells play a critical role in the activation and recruitment of neutrophils [23]. Our previous studies have shown that exposure to EPFRs results in maturation of dendritic cells and Th17 biased cell responses that were associated with neutrophilic inflammation [35]. However, the mechanism of EPFR-induced Th17 inflammation and associated neutrophilia was not completely understood. In the current study, MCP230-exposure caused an early immune response in the form of increased Th17 cytokines including neutrophil chemo-attractants. Using IL23p19 knockout (IL23p19^−/−^; mice deficient in Th17 responses) and IL17Ra knockout (IL17Ra^−/−^) mice models, we have confirmed that activation of Th17 cells and IL17A are essential for neutrophilic inflammation in the lungs upon exposure to EPFRs.

AHR binds to a wide array of exogenous and endogenous ligands, activating inflammatory responses in various diseases [27]. AHR is known to play a role in increased differentiation of Th17 cells [30, 31]. Th17 cell differentiation subsequent to ligand-dependent activation of AHR has also been observed in other models of autoimmune diseases [29–31], which may lead to exacerbation of inflammation involving neutrophilia. To understand the role of AHR in MCP230-induced Th17 inflammation, we investigated whether acute exposure to MCP230 activates the AHR pathway. Our results showed that exposure to EPFRs induced activation of AHR (Fig. 3) and was confirmed by increased expression of *Cyp1a1* and *Cyp1b1* in lung cells in vitro (Figs. 3, 4 and 5) and in vivo (Fig. 6). This EPFR-induced *Cyp1a1* and *Cyp1b1* expression was inhibited by an AHR antagonist (Fig. 5). Previously, we reported that EPFR-induced oxidative stress and cellular uptake of MCP230 particles are important for its immune-modulating effects [35]. Oxidative stress is known to promote the polarization of T cell differentiation towards Th2 phenotype [48]; however, the role of oxidative stress in polarizing the T cell differentiation towards Th17 phenotype is not completely understood and in the literature there is conflicting information on the induction of oxidative stress as a result of AHR activation [49, 50]. Interestingly, our data showed that using an antioxidant or an uptake blocker significantly inhibited the EPFR-induced *Cyp1a1* and *Cyp1b1* expression (Fig. 5), suggesting a role of EPFR-induced oxidative stress in AHR activation.

IL17 mediates the influx of neutrophils in the lungs in an OVA-induced mouse model of asthma and inhibiting IL17, using monoclonal antibodies, reduces the bronchial influx of neutrophils [51]. In addition, Th17 cells can induce neutrophilia by directly releasing chemoattractants for neutrophils [25]. We demonstrate that exposure to EPFRs results in increased levels of Th17 inducing cytokines IL1β and IL6 levels at 1 dpe, with significantly high levels of IL6 as early as 12 h post-exposure. IL6 is an important regulator of balance between regulatory T cells and Th17 cells [52]. The levels of IL33 were also higher at 12 h post-exposure and 1 dpe, which can directly stimulate mast cells to produce IL1β and IL6, and enhance the Th17 response [53, 54]. Further, we observed increase in cytokines IL17A and an increase in the levels of neutrophil chemo attractants KC and MCP-1 in the airways at 1 dpe. Although we observed only a transient activation of AHR, such AHR activation can drive cytokine induction that leads to Th17 biased responses. We observed EPFR-induced immune responses in mice beginning at 12 h post-exposure that persisted until 5 dpe. Further studies are required to understand the role of AHR activation in early immune response and its effects on subsequent adaptive Th17 response.

We present evidence that AHR activation is necessary for EPFR-induced pulmonary immune responses. Treatment with AHR antagonist resulted in suppression of EPFR-induced pulmonary Th17 responses and the associated neutrophilic inflammation. AHR is expressed at high levels in Th17 cells compared to other T cell subsets [28] and we observed a significant reduction of EPFR-induced Th17 cells in mice pre-treated with AHR antagonist.

## Conclusions

We demonstrated that EPFRs associated with combustion-derived PM result in pulmonary immune response in the form of early Th17 cytokine expression and subsequent pulmonary neutrophilic inflammation. This EPFR-induced pulmonary neutrophilic inflammation was shown to be dependent on Th17 and IL17A. Our in vitro data demonstrated that EPFR-induced AHR activation is mediated to some extent by the ability of particles to generate oxidative stress and required uptake of EPFRs. Further, we demonstrated that EPFRs induce AHR activation in vivo and inhibition of AHR activation using selective AHR antagonist resulted in inhibition of pulmonary Th17 inflammation and associated neutrophilia. In summary, our data illustrate a unique mechanism by which radical containing particulate matter mediate pulmonary Th17 immune responses through AHR. In addition to Th17 cells, other cells like γδ T cells [55, 56] and NKT cells [57] can produce IL17A and contribute to inflammation prior to the development of adaptive Th17 responses. Although beyond the scope of this paper, our future studies will investigate the role of AHR activation in these cells. Using Th17^−/−^ mouse model would directly address the importance of Th17 cells in MCP230-exacerbated asthma severity; however, this model was not available when our experiments were performed. While AHR−/− mice were available at the time of these studies, we chose not to use them, because among other physiological issues (e.g. defects in fertility, perinatal growth, liver size and function, closure, spleen size), they have defects in peripheral lymphocytes [58], which would be important in the inflammatory responses to MCP230. Generation of conditional and cell-specific AHR^−/−^ models will be helpful to confirm our results, but beyond the scope of this manuscript.

## Additional files

Additional file 1: **Figure S1.** *Il17f* expression relative to *Gapdh* in whole-lung homogenates of WT and IL23p19^−/−^ mice exposed to vehicle or MCP230 was determined using TaqMan gene expression assay (Applied Biosystems, Waltham, MA). Expression was determined at 5 dpe. Data represent mean ± SEM from 3 to 5 mice. (TIF 148 kb)

Additional file 2: **Figure S2.** WT and IL17Ra^−/−^ mice were sensitized with OVA (ovalbumin complexed to Imject Alum) on days 0 and 14. Mice were exposed to either vehicle or MCP230 (50 μg) on protocol day 23 and challenged with OVA on days 24, 25, and 26. BAL fluid or lungs were collected on day 28. Differential cell counts of BALF cells at 5 dpe (i.e. day 28) from mice challenged with OVA and exposed to vehicle or MCP230. Data are presented as mean ± SEM of numbers of cells from 4 to 5 mice. (TIF 9277 kb)

## Abbreviations

AHR: Aryl hydrocarbon receptor
ANOVA: Analysis of variance
BALF: Bronchoalveolar lavage fluid
BMDC: Bone marrow-derived dendritic Cell
CH223191: 1-methyl-*N*-[2-methyl-4-[2-(2-methylphenyl)diazenyl]phenyl-1*H*-pyrazole-5-carboxamide)
*Cyp1a1*: Cytochrome p450 1a1
*Cyp1b1*: Cytochrome p450 1b1
EPFR: Environmentally persistent free radicals
FICZ: 6-formylindolo[3,2-b]carbazole
IL17A: Interleukin 17A
IL17E: Interleukin 17E
IL1β: Interleukin 1β
IL21: Interleukin 21
IL22: Interleukin 22
IL31: Interleukin 31
IL33: Interleukin 33
IL6: Interleukin 6
KC: Keratinocyte-derived chemokine
MCP-1: Monocyte chemotactic protein-1
MCP230: EPFR-containing PM
OVA: Ovalbumin
PBN: *N*-tert-Butyl-α-phenylnitrone
PM: Particulate matter
SEM: Standard error of means
TCDD: 2,3,7, 8-tetrachlorodibenzo-p-dioxin
Th17: T-helper 17
