# Supplementary figures and images for: Radical containing combustion derived particulate matter enhance pulmonary Th17 inflammation via the aryl hydrocarbon receptor
Source: Part Fibre Toxicol. 2018 May 3;15:20. doi: 10.1186/s12989-018-0255-3 (PMC5934866; doi:10.1186/s12989-018-0255-3)

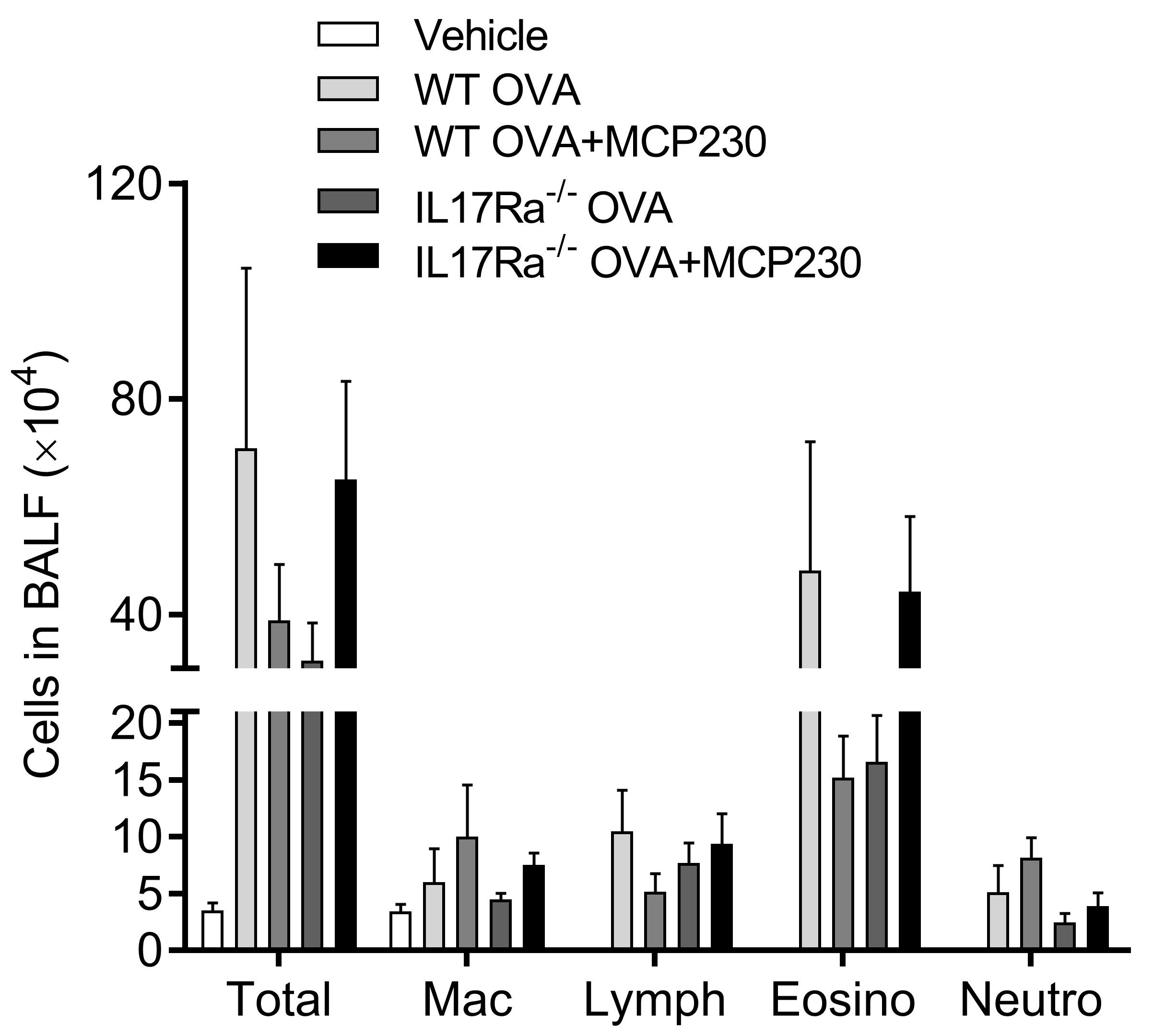

Supplement: Supplementary file 1 — Figure S1. Il17f expression relative to Gapdh in whole-lung homogenates of WT and IL23p19−/− mice exposed to vehicle or MCP230 was determined using TaqMan gene expression assay (Applied Biosystems, Waltham, MA). Expression was determined at 5 dpe. Data represent mean ± SEM from 3 to 5 mice. (TIF 148 kb) [file 12989_2018_255_MOESM2_ESM.tif]

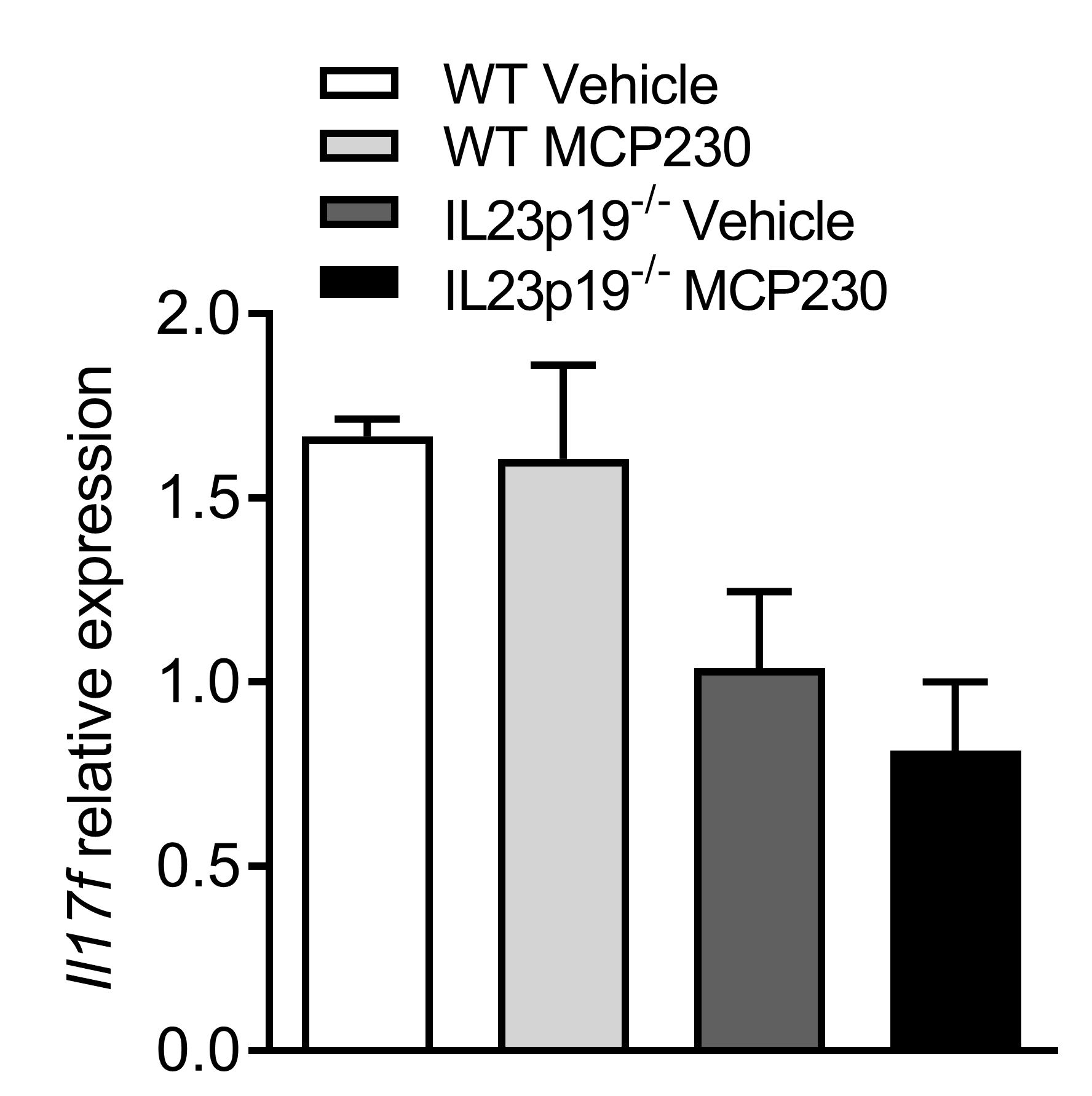

Supplement: Supplementary file 2 — Figure S2. WT and IL17Ra−/− mice were sensitized with OVA (ovalbumin complexed to Imject Alum) on days 0 and 14. Mice were exposed to either vehicle or MCP230 (50 μg) on protocol day 23 and challenged with OVA on days 24, 25, and 26. BAL fluid or lungs were collected on day 28. Differential cell counts of BALF cells at 5 dpe (i.e. day 28) from mice challenged with OVA and exposed to vehicle or MCP230. Data are presented as mean ± SEM of numbers of cells from 4 to 5 mice. (TIF 9277 kb) [file 12989_2018_255_MOESM1_ESM.tif]
